# Supplementary material for: Downregulation of basal myosin‐II is required for cell shape changes and tissue invagination
Source: EMBO J. 2018 Nov 15;37(23):e100170. doi: 10.15252/embj.2018100170 (PMC6276876; doi:10.15252/embj.2018100170)
Supplement: Supplementary file 1 — Expanded View Figures PDF [file EMBJ-37-e100170-s001.pdf]

Expanded View Figures

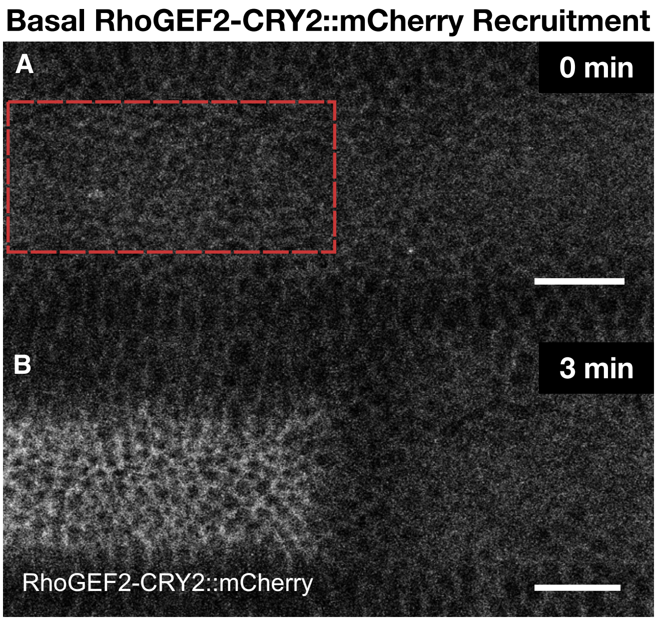

Figure EV1. Light-mediated recruitment of RhoGEF2-CRY2 to the plasma membrane.

A, B Embryos co-expressing CIBN::GFPpm and RhoGEF2-CRY2::mCherry were mounted with their dorsal epithelium facing the objective, and a region of interest (red dashed line) (A) was defined to specifically illuminate the cell base at ~25 μm from the apical surface in a subset of the embryonic tissue using two-photon microscopy. A representative experiment showing RhoGEF2-CRY2::mCherry from top view (sum-of-slice projection of 5 focal planes) at the initial stage of the experiment (A) and 3 min after activation (B). RhoGEF2-CRY2::mCherry localized to the cytoplasm before activation and was specifically recruited to the plasma membrane upon light activation. Scale bar, 20 μm.

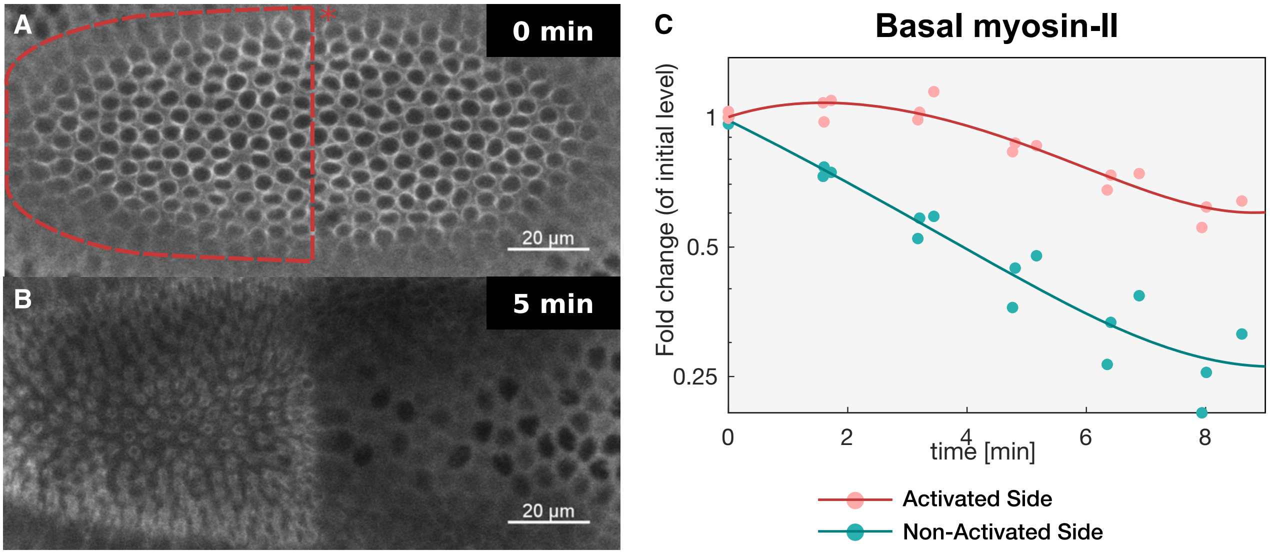

Figure EV2. Light-mediated recruitment of RhoGEF2-CRY2 to the plasma membrane increases myosin-II levels at the basal surface of ventral cells.

A, B Embryos co-expressing CIBN::GFPpm/RhoGEF2-CRY2 and the myosin-II probe Sqh::mCherry were mounted with the ventral tissue facing the objective, and a region of interest (red dashed line) (A) was defined to specifically illuminate the cell base at ~25 μm from the apical surface in a subset of the embryonic tissue using two-photon microscopy. Note that due to the curvature of the embryo, a half-elliptic region of activation was defined to avoid activation of adjacent lateral cells. A representative experiment (N = 8) showing basal Sqh::mCherry from top view (sum-of-slice projection of 5 focal planes) at the beginning of the experiment (A) and 5 min after activation (B). Sqh::mCherry levels were stabilized in the region of activation while dropped in the non-activated tissue.

C Quantification of myosin-II levels (N = 3) in the activated region (red) and non-activated region (green). While myosin-II levels progressively decreased in the non-activated region, myosin-II depletion slowed down in the activated region. The straight line indicates a polynomial fit (3rd degree) to the respective data (r^2 = 0.91 for the activated region and r^2 = 0.97 for the non-activated region). Note the logarithmic y-axis scale.

© 2018 European Molecular Biology Laboratory

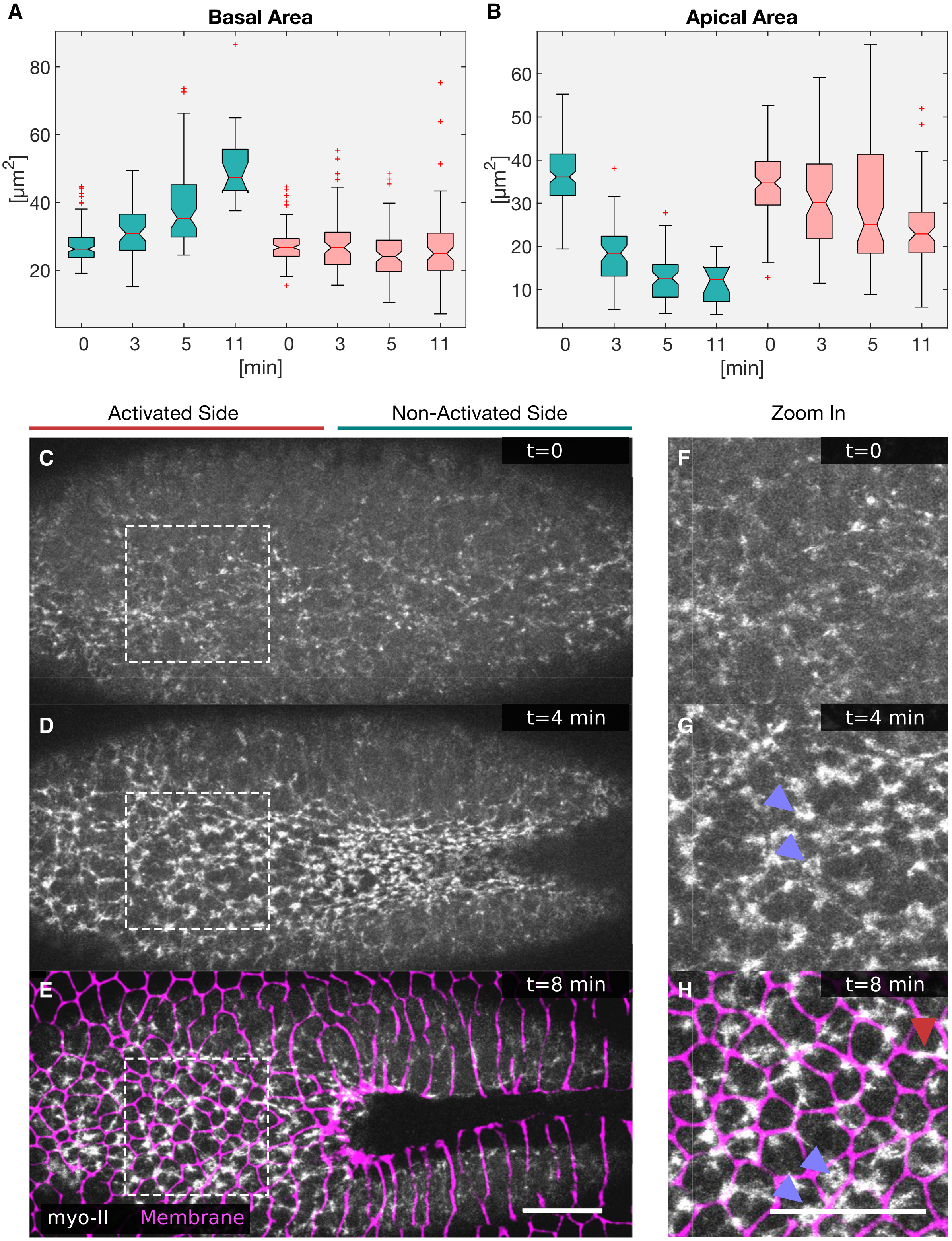

Figure EV3.

 © 2018 European Molecular Biology Laboratory

Figure EV3. Stabilization of basal myosin-II inhibits apical constriction and basal expansion.

A, B Cells were photo-activated at the cell base, and the cell shapes reconstructed to extract and quantify specific features. (A) The basal surface increased in the non-activated region and was kept constant in the activated region. (B) While in the non-activated region the apical area decreased progressively, apical constriction was inhibited in the activated region (red). In each box plot, the central mark, the bottom, and the top edge of each box indicate the median, the 25th percentile, and 75th percentile, respectively. Whiskers extent to the most extreme data point, and the "+" symbol indicates an outlier. Notches indicate comparison intervals.

C–H *Drosophila* embryos expressing the optogenetic module CIBN::GFPpm/RhoGEF2-CRY2 and the myosin-II probe Sqh::mCherry were mounted with the ventral tissue facing the objective. The anterior half of the embryo was activated at the cell base, and the Sqh::mCherry signal was recorded in a 5-µm-sized image stack. Top view showing apical myosin-II distribution at the initial time point (C), 4 min (D), and 8 min (E) after initial activation. (F–H) Apical myosin-II distribution in the activated region at the initial time point (F), 4 min (G), and 8 min (H) after initial activation in high magnification of the regions indicated by white dashed square in (C–E). Myosin-II accumulated in both the non-activated and activated region. (G–H) In the activated region, myosin-II accumulated in the center of the cells, in stable ring-like structures (blue arrowheads), or to cell junctions (red arrowhead). (E–H) Immediately after the final Sqh::mCherry acquisition, the plasma membrane signal (CIBN::GFPpm, in magenta) was recorded and superimposed to the myosin-II signal. Scale bars, 25 µm.

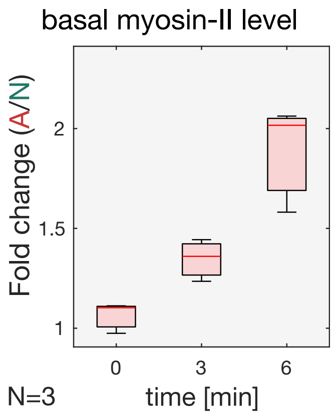

Figure EV4. Light-mediated recruitment of RhoGEF2-CRY2 to the basal-specific anchor PatJ-CIBN::GFPpm increases myosin-II levels at the basal surface of ventral cells.

Embryos co-expressing CIBN-PatJ::GFPpm, RhoGEF2-CRY2, and the myosin-II probe Sqh::mCherry were mounted with the ventral tissue facing the objective. The cell base was illuminated in a subset of the embryonic tissue using two-photon illumination when cells were already apically constricted and elongated, and the ventral tissue was already internalized. The Sqh::mCherry signal was recorded prior to photo-activation, 3 and 6 min thereof. Boxplot shows the fold change of myosin-II levels ($N = 3$) in the activated compared to the non-activated region. After 6 min of photo-activation, myosin-II levels were ~2-fold higher in the photo-activated region than in the non-photo-activated area. In each box plot, the central mark, the bottom, and the top edge of each box indicate the median, the 25th percentile, and 75th percentile, respectively. Whiskers extent to the most extreme data point.

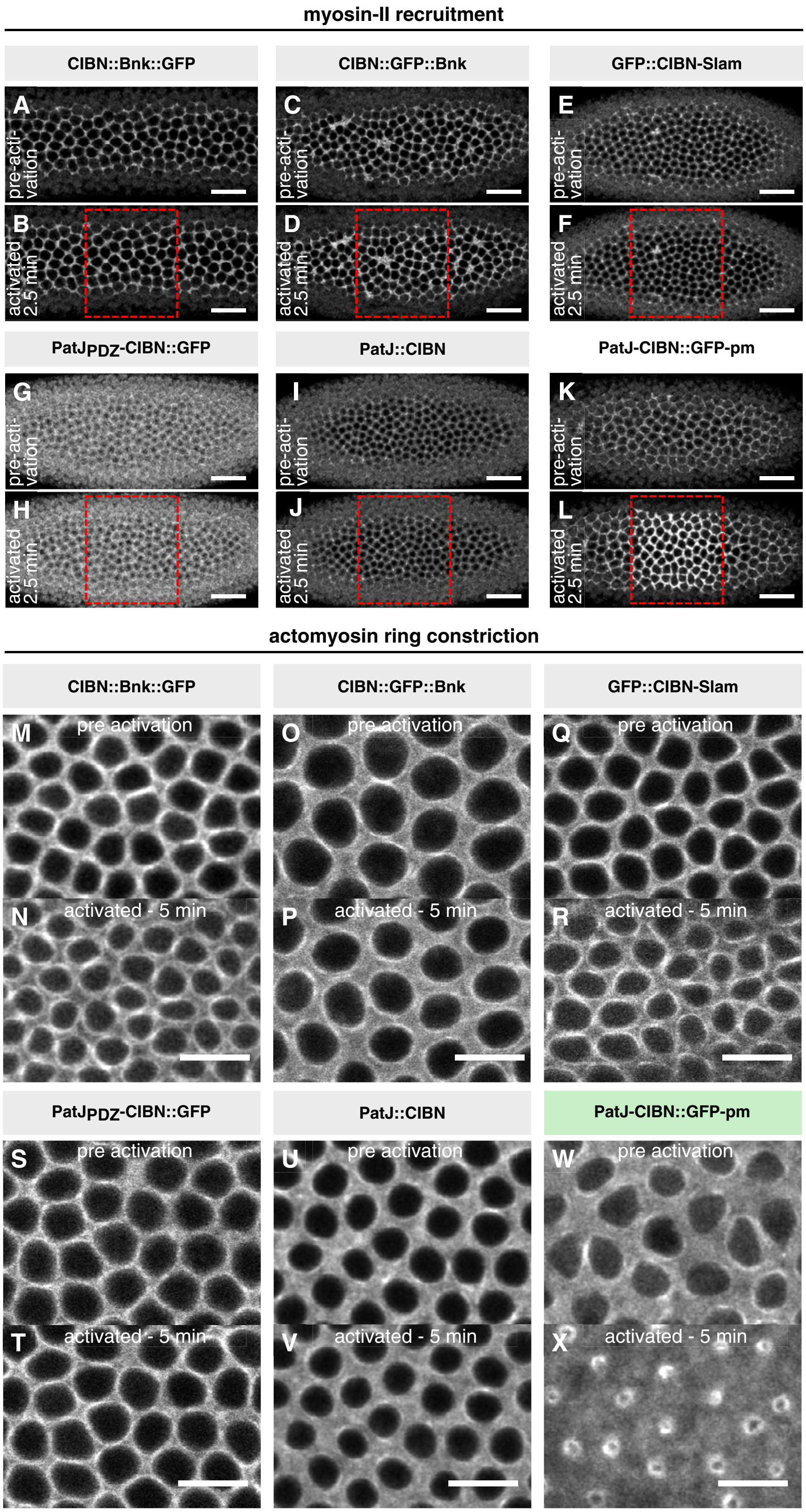

Figure EV5. Among the tested basal anchors, only CIBN-PatJ::GFPpm supports myosin-II plasma membrane recruitment upon RhoGEF2-CRY2 photo-activation.

A–L Embryos co-expressing RhoGEF2-CRY2, Sqh::mCherry, and the indicated basal-specific anchors (A,B: CIBN::Bnk::GFP; C, D: CIBN::GFP::Bnk; E, F: GFP-CIBN-Slam; G, H: PatJPDZ-CIBN::GFP; I, J: PatJ-CIBN; K, L: PatJ-CIBN::GFPpm) were photo-activated using one photon illumination (488 nm) within a region of interest (red dashed box). Myosin-II signal (Sqh::mCherry) was recorded prior and after photo-activation. Representative image ($N \geq 2$) showing the initial actomyosin network before (A, C, E, G, I, and K) and 2.5 min after photo-activation (B, D, F, H, J, and L). Scale bar, 20 μm.

M–X Embryos co-expressing the indicated basal anchors, RhoGEF2-CRY2 and Sqh::mCh were photo-activated using one photon illumination (488 nm), and Sqh::mCh was recorded simultaneously to visualize myosin-II recruitment and effects on the actomyosin network during cellularization. The upper panels (M, O, Q, S, U, W) show the basal actomyosin network before photo-activation and the lower panels (N, P, R, T, V, X) 5 min after continuous photo-activation demonstrating that only in the presence of CIBN-PatJ::GFPpm, actomyosin ring constricted. Scale bar, 10 μm.

© 2018 European Molecular Biology Laboratory
