# Supplementary material for: Downregulation of basal myosin‐II is required for cell shape changes and tissue invagination
Source: EMBO J. 2018 Nov 15;37(23):e100170. doi: 10.15252/embj.2018100170 (PMC6276876; doi:10.15252/embj.2018100170)
Supplement: Supplementary file 2 — Movie EV1 [file EMBJ-37-e100170-s002.zip › EMBOJ-2018-100170_MovieEV1.pdf]

Movie EV1. Cross section showing myosin-II during ventral furrow formation.

Embryos were mounted vertically with the posterior pole facing the objective to record and visualize the transverse cross section of the embryonic tissue. Representative embryo (N=4) expressing the myosin-II marker Sqh::GFP imaged by two-photon microscopy. Scale bar, 40 μm.
