# Supplementary material for: Downregulation of basal myosin‐II is required for cell shape changes and tissue invagination
Source: EMBO J. 2018 Nov 15;37(23):e100170. doi: 10.15252/embj.2018100170 (PMC6276876; doi:10.15252/embj.2018100170)
Supplement: Supplementary file 3 — Movie EV2 [file EMBJ-37-e100170-s003.zip › EMBOJ-2018-100170_MovieEV2.pdf]

Movie EV2. Top view showing basal myosin-II distribution upon basal photo-activation in the dorsal tissue.

Embryos co-expressing the Rho-GEF2-Cry2/CIBN::GFP optogenetic module and the myosin-II marker Sqh::mCherry were mounted with the dorsal tissue facing the objective. A Sqh::mCherry image stack was recorded in alternation with photo-activation of the base of a subset of cells (red box). Top view (sum of 6 slices) showing the basal myosin-II distribution. Scale bar, 20 μm.
