# Supplementary material for: Downregulation of basal myosin‐II is required for cell shape changes and tissue invagination
Source: EMBO J. 2018 Nov 15;37(23):e100170. doi: 10.15252/embj.2018100170 (PMC6276876; doi:10.15252/embj.2018100170)
Supplement: Supplementary file 4 — Movie EV3 [file EMBJ-37-e100170-s004.zip › EMBOJ-2018-100170_MovieEV3.docx]

**Movie EV3. Cross section showing myosin‑II distribution upon basal photo-activation in the dorsal tissue.**

Embryos co-expressing the Rho-GEF2-Cry2/CIBN::GFP optogenetic module and the myosin-II marker Sqh::mCh were mounted with the dorsal tissue facing the objective. Z-projection of Sqh::mCherry image stacks were recorded in alternation with photo-activation of the base of a subset of cells (red box). Scale bar, 20 µm.
