# Supplementary material for: Downregulation of basal myosin‐II is required for cell shape changes and tissue invagination
Source: EMBO J. 2018 Nov 15;37(23):e100170. doi: 10.15252/embj.2018100170 (PMC6276876; doi:10.15252/embj.2018100170)
Supplement: Supplementary file 5 — Movie EV4 [file EMBJ-37-e100170-s005.zip › EMBOJ-2018-100170_MovieEV4.docx]

**Movie EV4. Apical myosin‑II distribution upon basal photo-activation during ventral furrow formation.**

Embryos co-expressing the Rho-GEF2-Cry2/CIBN::GFP optogenetic module and the myosin-II marker Sqh::mCherry were mounted with the ventral tissue facing the objective. A Sqh::mCh image stack was recorded in alternation with photo-activation of the cell base of the anterior side of the embryo (indicated by the red line). Top view (sum of 10 slices) showing the apical myosin-II distribution. Note that the non-activated cells internalize and disappear from the field of view. Scale bar, 20 µm.
