# Supplementary material for: Downregulation of basal myosin‐II is required for cell shape changes and tissue invagination
Source: EMBO J. 2018 Nov 15;37(23):e100170. doi: 10.15252/embj.2018100170 (PMC6276876; doi:10.15252/embj.2018100170)
Supplement: Supplementary file 6 — Movie EV5 [file EMBJ-37-e100170-s006.zip › EMBOJ-2018-100170_MovieEV5.pdf]

Movie EV5. Apical pulsatile contractions upon basal photo-activation during ventral furrow formation.

Embryo co-expressing the Rho-GEF2-Cry2/CIBN::GFP optogenetic module and the membrane marker GAP43::mCherry were mounted with the ventral tissue facing the objective. An apical 5 μm-sized GAP43::mCherry image stack was recorded in alternation with photo-activation (20 s) of the anterior side of the cell base of the embryo (red line). The total time resolution for the GAP43::mCh acquisition was 35 sec. Cells were segmented and tracked over time using EDGE software. Green-colored cells indicate non-activated cells; red- and blue-colored cells indicate constricting and non-constricting cells, respectively, in the activated region. Scale bar, 20 μm.
